# WIP Regulates the Stability and Localization of WASP to Podosomes in Migrating Dendritic Cells

**DOI:** 10.1016/j.cub.2006.10.037

**Published:** 2006-12-05

**Authors:** Hsiu-Chuan Chou, Inés M. Antón, Mark R. Holt, Claudia Curcio, Stefania Lanzardo, Austen Worth, Siobhan Burns, Adrian J. Thrasher, Gareth E. Jones, Yolanda Calle

**Affiliations:** 1 Randall Division of Cell & Molecular Biophysics, King's College London, London SE1 1UL, United Kingdom; 2 Centro de Biología Molecular “Severo Ochoa”, CSIC-UAM, 28049 Madrid, Spain; 3 Department of Clinical and Biological Sciences, University of Turin, 10043 Orbassano, Turin, Italy; 4 Molecular Immunology Unit, Institute of Child Health, University College London, London and GOSH NHS Trust WC1 1EH, United Kingdom

**Keywords:** CELLBIO, MOLIMMUNO

## Abstract

The Wiskott-Aldrich Syndrome protein (WASP) is an adaptor protein that is essential for podosome formation in hematopoietic cells [1]. Given that 80% of identified Wiskott-Aldrich Syndrome patients result from mutations in the binding site for WASP-interacting-protein (WIP) [2], we examined the possible role of WIP in the regulation of podosome architecture and cell motility in dendritic cells (DCs). Our results show that WIP is essential both for the formation of actin cores containing WASP and cortactin and for the organization of integrin and integrin-associated proteins in circular arrays, specific characteristics of podosome structure. We also found that WIP is essential for the maintenance of the high turnover of adhesions and polarity in DCs. WIP exerts these functions by regulating calpain-mediated cleavage of WASP and by facilitating the localization of WASP to sites of actin polymerization at podosomes. Taken together, our results indicate that WIP is critical for the regulation of both the stability and localization of WASP in migrating DCs and suggest that WASP and WIP operate as a functional unit to control DC motility in response to changes in the extracellular environment.

## Results and Discussion

Cell migration requires the rearrangement of adhesions and cytoskeleton in response to extracellular stimuli to form a leading edge that allows directed locomotion. In most cell types, migration-related adhesive structures are composed of integrin heterodimers and intracellular associated proteins clustered in plaques termed focal complexes and their more mature variant, focal adhesions [3]. In addition to these adhesive structures, DCs also assemble distinctive adhesions named podosomes that are characteristic of cells of the myeloid lineage including macrophages and osteoclasts 4, 5. The molecular composition of podosomes is similar to that of focal complexes and focal adhesions. However, they have a unique organization with actin filaments bundled in discrete foci forming a conical core containing distinctive elements such as gelsolin, cortactin, the actin-nucleating factor Arp 2/3 complex, and the scaffolding protein WASP [4] surrounded by a ring of integrins and integrin-associated proteins 4, 5.

WASP is an essential component of podosomes, where it activates the Arp2/3 complex inducing actin polymerization [6]. WIP has also been detected in the core of podosomes in endothelial cells expressing eGFP-WIP [7], and although there is evidence indicating that WIP plays a major role in regulating the activity of WASP family proteins and the dynamics of actin filaments 8, 9, it remains unknown how WIP participates in the assembly of podosomes.

### WIP Is Required for the Assembly of Actin Filaments, β2-Integrins, and Associated Proteins into Podosome Complexes

**Figure 1:**
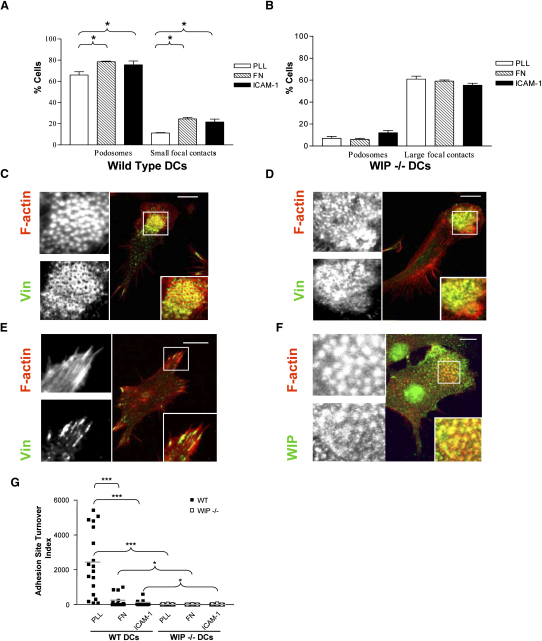
WIP Is Required for Podosome Formation (A and B) DCs plated on poly-L-lysine (PLL), fibronectin (FN), or intercellular cell adhesion molecule-1 (ICAM-1)-coated glass coverslips were double-stained to identify the distribution of vinculin and F-actin to allow the quantification of the percentage of podosomes and focal contacts in wild-type (A) and WIP^−/−^ (B) DCs. Graphs illustrate the mean percentage of cells with podosomes ± SE of four experiments (n = 80 cells per experiment). Unpaired Student's t test was used to assess the significance of the difference between cell types. ^∗^p < 0.05. (C–E) Confocal micrographs showing the distribution of F-actin (red) and vinculin (green) in DCs plated on PLL. About 70% of wild-type DCs assembled podosomes with well-defined actin cores and vinculin rings (C). Only about 7% of WIP^−/−^ DCs assembled podosomes that displayed abnormally fused actin cores and irregularly formed vinculin rings (D). Large focal contacts were assembled in about 60% of WIP^−/−^ cells (E), replacing podosomes as the most prominent adhesive structure. Magnifications of the boxed areas in (C), (D), and (E) with F-actin and vinculin staining are shown on the left and merged on the bottom right. (F) Distribution in wild-type DCs of WIP (green) and actin (red). Magnifications of the boxed areas in (F) with WIP and F-actin staining are shown on the left and merged on the bottom right. Scale bars represent 10 μm. (G) Graph illustrating the adhesion site turnover index calculated as described in Supplemental Experimental Procedures. As shown, WIP^−/−^ DCs develop highly stable adhesions compared to wild-type DCs. The graph illustrates the combined results from at least three independent experiments. The gray line indicates the mean value of each adhesion turnover index. ^∗^p < 0.05, ^∗∗∗^p < 0.001; unpaired Student's t test.

To study the role of WIP in podosome formation, the distribution of F-actin and vinculin was examined in wild-type and WIP^−/−^ DCs by confocal microscopy. As expected, podosomes were grouped in clusters in wild-type DCs (Figures 1A and 1C). In contrast, very few WIP^−/−^ DCs formed podosomes (Figure 1B). Those few podosomes formed in WIP^−/−^ DCs displayed an abnormal structure having less discrete actin cores and disorganized vinculin rings around F-actin (Figure 1D). Large focal contacts containing vinculin comprised the most striking adhesion structures in WIP^−/−^ DCs (Figures 1B and 1E), replacing podosomes as the major points of contact with the substratum. The critical involvement of WIP in DC podosome formation is also supported by the presence of endogenous WIP in the core of podosomes in wild-type cells (Figure 1F).

Our data suggest that the key role of WIP in podosome formation is in the organization of F-actin and the clustering of integrins and associated proteins. The absence of WIP had a profound effect on the organization of F-actin within DCs. While the bulk of F-actin in wild-type DCs was associated with the core of podosomes (Table 1, see Figure S1A in the Supplemental Data available online), in WIP^−/−^ DCs it distributed among various anomalous structures including actin cables (Table 1, Figure S1B), large fused actin aggregates, commonly with a circular shape (Table 1, Figure S1C), and extensive membrane ruffles (Table 1, Figure S1D). Clustering of podosomal integrins and the integrin-associated proteins paxillin and talin was also affected. In wild-type DCs, β2-integrins formed rings around the F-actin cores of podosomes (Figure S2A) as previously described [10]. In contrast, WIP^−/−^ DCs failed to cluster β2-integrins into rings, and instead they formed small integrin-containing focal contacts scattered through the basal surface and around the periphery of the cell (Figure S2B) or large plaques associated with anomalous actin-rich aggregates (Figure S2C). FACS analysis showed that wild-type and WIP^−/−^ DCs expressed equivalent levels of β1- and β2-integrins (Figure S3), indicating that WIP does not affect integrin expression. Paxillin and talin, which normally array around the podosome core in wild-type DCs [11] (Figures S2D and S2G), in WIP^−/−^ DCs localized to focal adhesions (Figures S2E and S2H) containing β1 integrins (data not shown) or formed amorphous clusters colocalizing with the anomalous actin aggregates (Figures S2F and S2I).

**Table 1:** Percentage of DCs with Actin Structures

	Podosomes	Actin Foci	Actin Cables	Actin Aggregates	Only Ruffles
WT DCs	68.7 ± 3.5	6.1 ± 2.3	4.3 ± 0.6	11.2 ± 1.3	9.8 ± 1.4
WIP^−/−^ DCs	6.7 ± 1.8	12.3 ± 1.0	19.4 ± 4.7	32.6 ± 9.9	29.4 ± 3.5

Analysis of the organization of actin filaments in wild-type (WT) and WIP^−/−^ DCs plated on PLL-coated glass coverslips and immunostained to determine the distribution of vinculin and F-actin via confocal microscopy. Data indicate the percentage of cells displaying podosomes (actin foci surrounded by a vinculin ring as determined by immunofluorescence), actin foci lacking vinculin rings, actin cables, and actin aggregates (amorphous plaques of actin filaments). Although some forms of actin aggregates were also detected in 10% of the wild-type DC population (compared to 32.6% in WIP^−/−^ DC cultures), these aggregates were qualitatively different. They appeared as clouds of actin with a lower amount of actin filaments (as detected by the lower intensity of phalloidin staining) compared to the WIP^−/−^ aggregates and the cells formed as major adhesions focal complexes rather than focal adhesions assembled in WIP^−/−^ DCs. Data show mean ± SE of three experiments (n = 75 cells per experiment).

### WIP Regulates Adhesion Turnover in DCs

The shift of adhesion composition and structure from podosomes (short half-life in the range of 30 s to 5 min [11]) to large focal contacts (long half life in the range of 30 to 60 min [12]) in WIP^−/−^ DCs had a negative impact on adhesion turnover (Figure 1G). As previously described [11], interference reflection microscopy (IRM) analyses showed podosomes in wild-type DCs rapidly assembled and disassembled, giving a high turnover index (Figure 1G). In WIP^−/−^ DCs, although multiple transient and unstable lamellar protrusions were developed at the cell periphery, the major adhesion sites in contact with the substratum were highly stable with a significantly lower turnover index compared to wild-type (Figure 1G). Interestingly, we found a decrease in podosome turnover in wild-type DCs plated on fibronectin (FN) and ICAM-1, indicating that integrin ligands promote podosome stability (Figure 1G). This stability of adhesion on integrin ligands correlates with an increase in the percentage of wild-type DCs with well-defined podosomes and small focal contacts (Figure 1A) and accumulation of podosomal F-actin, integrins, and associated proteins (data not shown). In contrast, no significant differences were detected in turnover index of adhesions between WIP^−/−^ DCs plated on different substrata (Figure 1G). The presence of integrin ligands failed to induce major changes in the composition of adhesions of WIP^−/−^ DCs (Figure 1B) except for an increase in the size of the focal adhesions. Taken together, our data indicate that WIP plays a key role in regulating DC adhesion structure and dynamics on biologically relevant substrata, processes crucial for the migratory capacity of cells.

### WIP Regulates Polarization in DCs in Cooperation with Cortactin

**Figure 2:**
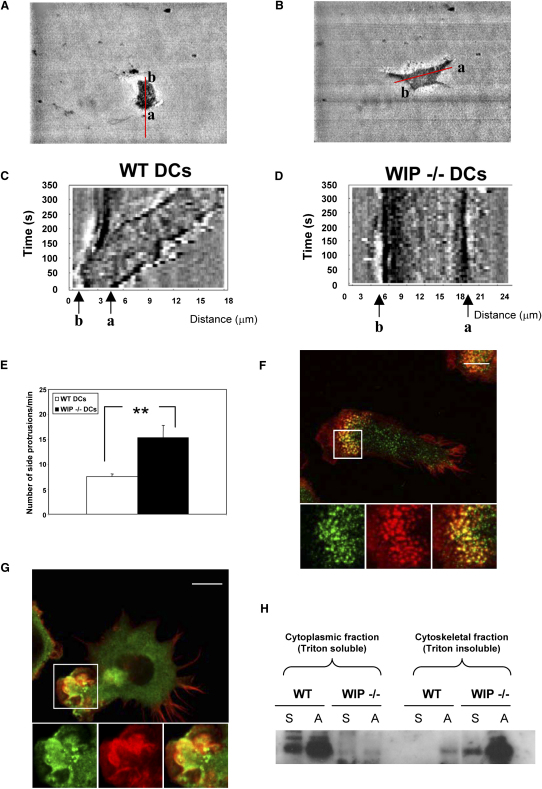
WIP Regulates DC Polarization (A–D) Kymographs obtained from IRM image stacks. Progression of the cell edges labeled “a” and “b” along the intensity line-scan drawn in the major axis of elongation of wild-type (A) and WIP^−/−^ (B) DCs are represented as kymographs in (C) and (D), respectively, with time on the y axis of the image and distance along the intensity profile on the x axis. While in wild-type DCs the edge labeled “a” became the leading edge and protruded in parallel with the retraction of the edge labeled “b” (C), edges in WIP^−/−^ DCs only produced unstable protrusions that assembled and collapsed continuously and did not result in a net progression of the edges (D). (E) The average frequency of lateral protrusions (along the short axis of elongated DCs; mean ± SE. ^∗∗^p < 0.01; unpaired Student's t test. (F and G) The distribution of cortactin (green) and F-actin (red) in wild-type and WIP^−/−^ DCs, respectively. Cortactin, F-actin staining, and merged images from the boxed areas in main panels are shown below. Confocal micrographs show the colocalization of cortactin with F-actin in the core of podosomes in wild-type DCs and the association with the multiple lateral ruffles in WIP^−/−^ DCs. Scale bar represents 10 μm. (H) The cytoplasmic (Triton soluble) and cytoskeletal (Triton insoluble) fractions of suspended (S) or adhered (A) wild-type (WT) or WIP^−/−^ DCs were subjected to SDS-PAGE and western blotted. Immunoblotting with an cortactin antibody shows an increase in the association of cortactin with the cytoskeletal fraction compared to WT DCs.

Stabilization of the leading edge in the direction of DC movement is provided by podosomes 13, 14. Migrating wild-type DCs clearly polarized, forming a patent leading edge, with the rear of the cell retracting at a similar rate, allowing net translocation of the cell body. In contrast, WIP^−/−^ DCs failed to develop a major leading front and instead formed multiple simultaneous and unstable lateral lamellae and ruffles (Figures 2A–2E; Movies S1 and S2). Cortactin is an Arp2/3 activator that plays a major role in the formation of podosomes 15, 16, establishment of cell polarization [17], and also generation of cellular cortical protrusions [18]. It has been proposed that cortactin synergises with the WASP family of proteins to induce actin polymerization [19], and it is thought that the actin filament meshwork triggered by the N-WASP/cortactin pair may have a different organization from actin meshworks triggered by N-WASP or cortactin alone [20]. Importantly, WIP has been shown to interact with cortactin and induce cortactin-mediated actin polymerization [21]. Our results show that in the absence of WIP, cortactin is displaced from podosome cores where it normally colocalizes with F-actin (Figure 2F) and is instead associated with random lamellar protrusions and ruffles over the cell periphery (Figure 2G). In addition, we found there was a higher proportion of cortactin associated with the cytoskeletal fraction (Triton insoluble) in adherent WIP^−/−^ DCs compared to wild-type DCs (Figure 2H). Taken together, these results show that in the absence of WIP, association of cortactin with the cytoskeleton is deregulated and associated with the formation of random cortical protrusions and loss of DC polarity. This suggests that the WASP/WIP complex may couple with cortactin to induce a type of meshwork of actin filaments and associated proteins that allow the clustering of β2-integrins during the assembly of podosomes and DC polarization. In the absence of WIP, cortactin cannot be recruited to form a complex with WASP, discrete actin foci in podosomes fail to form, and instead, cortactin codistributes with actin filaments at the cell margin, resulting in the random protrusions and reduced motility (data not shown) observed in WIP^−/−^ DCs.

### WIP Protects WASP from Degradation by Calpain in DCs

**Figure 3:**
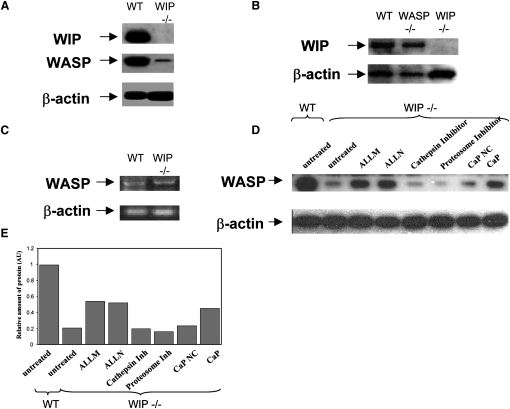
WIP Protects WASP from Degradation by Calpain in DCs (A and B) Total lysates of wild-type (WT) and WIP^−/−^ DCs were resolved by 12% SDS-PAGE, western blotted, and probed with WASP, WIP, or β-actin antibodies after stripping according to the experiment. β-actin showed the loading control in each lane. Expression of WASP was severely suppressed in WIP^−/−^ DCs (A), whereas similar amounts of WIP were expressed in WASP^−/−^ DCs (B). (C) To further dissect the basis of WASP expression in the absence of WIP, RT-PCR was performed to examine the mRNA levels of WASP in WIP^−/−^ cells. Levels of WASP mRNA were equivalent in WT and WIP^−/−^ DCs. (D) Inhibition of calpain with the calpain inhibitors ALLM, ALLN, and the peptide coding for the endogenous inhibitor of calpain, calpastatin (CaP), but not with proteosome or cathepsins inhibitors or the scrambled version of the calpastatin peptide (CaP NC) resulted in a major increase in WASP expression in WIP^−/−^ DCs. (E) Graph shows the quantification of the expression of relative amounts of WASP detected by immunoblot as shown in (D). The intensity of luminosity (I. L.) and the area of each band in an inverted image was calculated with the histogram function of Photoshop 7.0, and the value of relative amount of protein (AU) was calculated as follows: I. L. × Area (WASP)/I. L. × Area (β actin).

We have previously shown that WASP^−/−^ DCs display equivalent anomalies in β2-integrin clustering [10] and focal contact formation to that observed in WIP^−/−^ DCs [11]. Also we find here that like WASP^−/−^ DCs, WIP^−/−^ DCs fail to polarize and form and stabilize consistent leading edges. These similarities between phenotypes suggest a connection between WASP and WIP deficiencies. Recently, it has been proposed that WIP contributes to the stabilization of WASP expression and the regulation of WASP-mediated actin rearrangement 8, 22. We find here that WIP^−/−^ DCs express low levels of WASP at the protein level (Figure 3A), as recently described for WIP^−/−^ T-lymphocytes [22]. Levels of WIP expression were not affected by the lack of WASP in WASP^−/−^ DCs (Figure 3B). Since mRNA levels of WASP in WIP^−/−^ DCs were comparable to those of wild-type DCs (Figure 3C), we deduce that WASP is degraded in the absence of WIP. The levels of expression of the WIP binding partners cortactin, Nck, and CrkL was not affected in WIP^−/−^ DCs (Figure S4).

It has been predicted that WIP may prevent the exposure of WASP family proteins to proteases, because the 25-residue WASP binding motif from WIP wraps around the WIP binding domain of N-WASP [2]. WASP, but not N-WASP, is a substrate for the protease calpain in platelets [23], and we have recently found that calpain also cleaves WASP in migrating DCs [11]. Our present results show that WIP prevents WASP cleavage by calpain in DCs since inhibition of this protease in WIP^−/−^ DCs resulted in major recovery of WASP expression (Figures 3D and 3E). This suggests that WIP may play a crucial role in regulating the accessibility of WASP for calpain cleavage, which takes place during dissolution of podosomes [11]. A role for WIP in the maintenance of WASP levels is also suggested by recent findings showing that in WAS patients, mutations that prevent WASP binding to WIP result in diminished expression of WASP 24, 25. We suggest that the low levels of WASP in these WAS patients may be the result of extensive calpain-mediated cleavage because of failure of WASP-WIP interaction.

### WIP Is Required for Localization of WASP to Forming Podosomes

**Figure 4:**
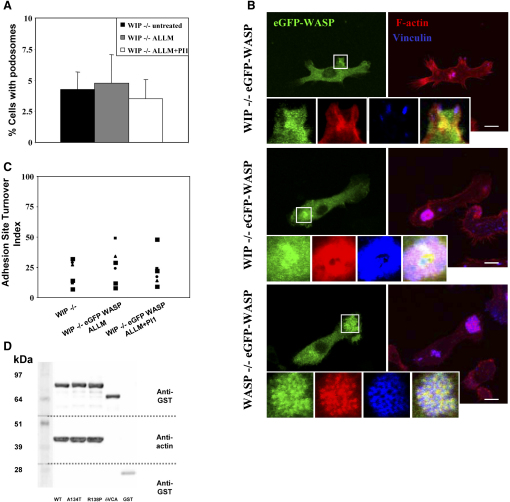
WIP Is Required for the Localization of WASP to Podosomes and the Recovery of the Dynamic Nature of Adhesions in DCs (A) The graph illustrates the mean percentage of cells with podosomes ± SE of four experiments (n = 25 cells per experiment). Recovery of WASP levels in WIP^−/−^ DCs treated with calpain inhibitors does not correlate with increased formation of podosomes. (B) Confocal micrographs show the distribution of eGFP-WASP (green), F-actin (red), and vinculin (blue) in WIP^−/−^ DCs (top and middle) and WASP^−/−^ DCs (bottom) expressing eGFP-WASP. Expression of eGFP-WASP in WASP^−/−^ DCs was achieved by infection of DCs with lentiviral vectors, resulting in reconstitution of podosomes where eGFP-WASP colocalized with actin in the core and was surrounded by vinculin forming a honeycomb array (see insets). Expression of similar levels of eGFP-WASP in WIP^−/−^ DCs required the treatment of cells infected with lentiviral vectors with calpain inhibitors and eGFP-WASP remained homogenously distributed in the cytoplasm or associated with amorphous actin structures (see insets). These actin aggregates fail to form the characteristic structure of podosomes. Vinculin was either absent from these actin aggregates (top) or was recruited forming an amorphous plaque (middle). Scale bars represent 10 μm. (C) The graph illustrates the distribution of adhesion turnover values of WIP^−/−^ DCs expressing eGFP-WASP by inhibiting calpain or calpain and the proteosome. No significant differences were detected in treated WIP^−/−^ DCs expressing eGFP-WASP compared to untreated WIP^−/−^ DCs. (D) Comparison of the ability of wild-type (WT), A134T, and R138P GST-tagged WASP recombinant protein, coated to sepharose beads to polymerize actin when incubated with U937 cell lysates. All three samples show similar levels of F-actin bound to the beads, while negative controls of GST only and δVCA WASP (WASP lacking the Arp 2/3 and actin-binding sites) demonstrate minimal actin binding.

Surprisingly, the recovery of WASP levels in WIP^−/−^ DCs by treatment with calpain inhibitors was not sufficient to induce podosome formation, and large focal contacts remained the major adhesion structures (Figure 4A). In addition, forced expression of eGFP-WASP in WIP^−/−^ DCs treated with calpain inhibitors alone or in conjunction with proteasome inhibitors [26] failed to restore podosome formation, and eGFP-WASP remained diffusely distributed in the cytoplasm or associated with anomalous F-actin aggregates that could contain vinculin forming abnormal plaques colocalizing with F-actin (Figure 4B, top and middle). Adhesion turnover remained as low as in WIP^−/−^ DCs (Figure 4C). This contrasts with the observed recovery of podosome formation and dynamics in WASP^−/−^ DCs (where WIP levels are normal) after the expression of eGFP-WASP (Figure 4B, bottom) 11, 13. We excluded the possibility that WIP binding is essential for the intrinsic actin polymerizing activity of WASP because specific WASP mutants (WASP A134T and WASP R138P) that interrupt WIP binding 24, 27 showed an actin polymerizing activity equivalent to wild-type WASP (Figure 4D). These results indicate that WIP not only protects WASP from calpain cleavage but also facilitates the localization of WASP to sites of actin polymerization and the organization of integrins, vinculin, and other integrin-associated proteins in circular clusters. The mechanisms by which WIP regulates WASP activity to allow podosome formation are yet to be fully elucidated. It is possible that WASP/WIP interactions may directly facilitate the recruitment of WASP to cell-surface receptors [28]. Alternatively, WIP may facilitate the bridging of WASP to these receptors by mediating the interaction with other adaptor proteins [29] or may regulate the function of regulatory proteins such as kinases, known to affect WASP activity 30, 31.

Our data show that WIP by itself is not sufficient to promote podosome assembly because WASP^−/−^ DCs, which express normal levels of WIP, also lack podosomes and assemble large focal contacts and disorganized actin clumps instead [4]. It is tempting to speculate that the cellular responses (of DC) to external soluble ligands such as chemoattractants would recruit WASP/WIP complexes coupled to integrin-associated proteins [32] to trigger the assembly of podosomes at appropriate sites in the developing leading edge of the cell [33]. These complexes would provide a cytoskeletal platform that regulates the accurate clustering and activation of β2-integrins [4], facilitating their function [34]. Taken together, our results suggest that WASP and WIP work as a functional unit in a cellular context as has been previously proposed 8, 9 to regulate podosome formation and dynamics during the establishment of cell polarity as seen in the chemotactic response to extracellular stimuli [35].

In summary, we have shown that in DCs WIP is essential for podosome formation and cell polarity by preventing the extensive degradation of WASP by calpain and by facilitating the recruitment of WASP to discrete foci to form the core of podosomes. The tight coupling between WASP and WIP is therefore a key contributor to normal podosome dynamics and likely of major importance to the cell trafficking behavior of DCs [36].

## Supplemental Data

Document S1. Four Figures and Supplemental Experimental Procedures

Movie S1. Interference Reflection Microscopy Films of Wild-Type DCs Plated on Poly-L-Lysine-Coated Glass Coverslips Assembled in Viewing Chambers Micrographs were taken 10 s apart and displayed at 10 frames per second. DCs formed highly dynamic podosomes that assembled and disassembled continuously behind the extending leading edge. DCs develop clear polarity with a well-defined leading edge and a retracting tail in the opposite pole.

Movie S2. Interference Reflection Microscopy Films of WIP^−/−^ DCs Plated on Poly-L-Lysine-Coated Glass Coverslips Assembled in Viewing Chambers Micrographs were taken 10 s apart and displayed at 10 frames per second. WIP^−/−^ DCs develop frequent highly unstable random protrusions and the major adhesions are highly stable. WIP^−/−^ DCs fail to develop effectively two functional migratory poles (leading edge and retracting tail).
