# Supplementary material for: WIP Regulates the Stability and Localization of WASP to Podosomes in Migrating Dendritic Cells
Source: Curr Biol. 2006 Dec 5;16(23):2337–44. doi: 10.1016/j.cub.2006.10.037 (PMC1885947; doi:10.1016/j.cub.2006.10.037)
Supplement: Document S1. Four Figures and Supplemental Experimental Procedures [file mmc1.pdf]

Supplemental Data

WIP Regulates the Stability and Localization of WASP to Podosomes in Migrating Dendritic Cells

Hsiu-Chuan Chou, Inés M. Antón, Mark R. Holt, Claudia Curcio, Stefania Lanzardo, Austen Worth, Siobhan Burns, Adrian J. Thrasher, Gareth E. Jones, and Yolanda Calle

Supplemental Experimental Procedures

Mice

$WIP^{-/-}$ mice from SV129/C57/BL6 mouse strain and SV129/C57/BL6 control (wild-type) mice were bred in the facilities of Universidad Autonoma de Madrid, Spain, as described [S1]. These mice were housed till 6–8 weeks old in the pathogen-free animal facility. $WASP^{-/-}$ mice on a SV129 background [S2] originally obtained from Dr. Fred Alt (Center for Blood Research, Harvard Medical School, Boston, MA) were bred in our colony in pathogen-free conditions.

Cell Culture

Individual spleens removed from 6- to 8-week-old mice after cervical dislocation were homogenized under sterile condition on ice by mechanical grinding with a syringe plunger through a 40 μm cell strainer. Cells were washed twice in PBS and then resuspended in RPMI supplemented with 10% FBS, 1 mM pyruvate (Sigma, UK), 1× nonessential aminoacids (Sigma), 2 mM glutamine (Sigma), 50 μm 2-ME (GIBCO-BRL, UK), 20 ng/ml recombinant mouse GM-CSF (R&D Systems, UK), and 1 ng/ml recombinant human TGF-β (R&D Systems) and plated at a density of 2 × 10^6 cells/ml in 75 cm^2 culture flasks at 37°C in a 5% CO_2 atmosphere. After 5 days of culture, 5 ml of fresh medium were added per flask, and at day 8, the cells in suspension were collected and kept in suspension in fresh medium. After a total of 17–18 days ex vivo, 80%–90% of the cells in culture were DCs as determined by the expression of CD11c by FACS analysis (data not shown). Cell viability prior to experimental assays was tested by Trypan blue exclusion.

Antibodies and Reagents

Antibodies were diluted in PBS containing 2.5% BSA as follows. (1) For immunofluorescence staining, anti-β1-integrin 1:250 (Pharmingen, UK); anti-β2-integrin 1:100 (Pharmingen); anti-Paxillin 1:100 (BD trans. Labs, UK); anti-Talin 1:100 (Sigma); anti-Vinculin 1:100 (Sigma); anti-Cortactin 1:100 (Sigma); Alexa 568-conjugated Phalloidin 1:100 (Molecular Probes, Eugene, OR); FITC-conjugated anti-Rat 1:100 (Sigma); anti-Hamster 1:100 (Jackson Immunoresearch Labs, West Grove, PA); Alexa 488-conjugated anti-Mouse IgG 1:100 (Molecular Probes); Cy5-conjugated anti-mouse IgG 1:100 (Jackson Immunoresearch Labs). An anti-WIP antiserum was raised by immunizing rabbits with a 19-amino acid C-terminal peptide of WIP (KLARNESRSGSNRRERGGP), which was used in the immunofluorescence studies at a concentration of 1:500. (2) For FACS staining, anti-β1-integrin 1:100 (Pharmingen); anti-β2-integrin 1:100 (Pharmingen); 1 μg FITC-conjugated anti-CD11c/10^6 cells (Pharmingen); anti-Paxillin 1:100 (BD trans. Labs); anti-Talin 1:100 (Sigma); anti-Vinculin 1:100 (Sigma); FITC-conjugated anti-rat 1:100 (Sigma); anti-hamster 1:100 (Jackson Immunoresearch Labs). (3) For western blots, anti-β-actin 1:1000 (Sigma); anti-WASP (aa 224–238) 1:1000 (Upstate Biotech, UK); anti-WIP (internal peptide) 1:500 (Santa Cruz Biotechnology, Santa Cruz, CA); anti-tubulin 1:2000 (Sigma);

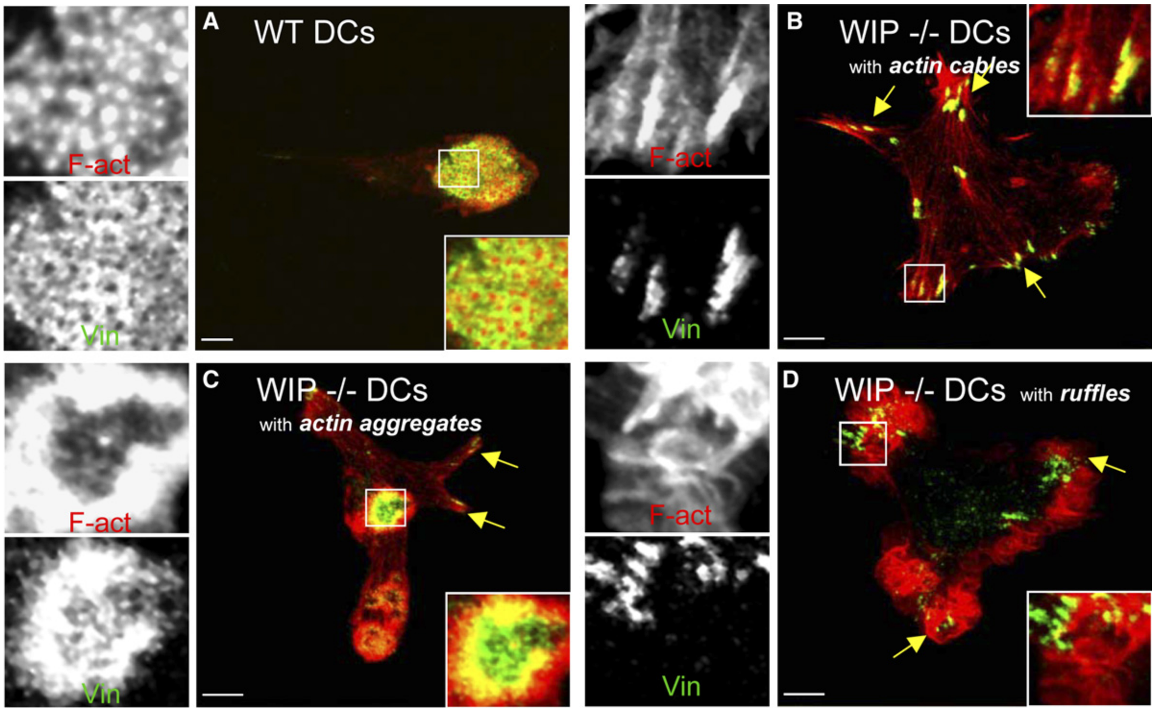

Figure S1. Organization of Actin Filaments in Wild-Type and $WIP^{-/-}$ DCs

Distribution of vinculin (green) and F-actin (red) in DCs plated on poly-L-lysine (PLL)-coated glass coverslips. Actin filaments in wild-type DCs were associated with podosomes (A) whereas in $WIP^{-/-}$ DCs actin filaments formed cables anchored by vinculin containing large focal contacts (yellow arrows) (B), amorphous aggregates (C), or extensive ruffles throughout the cell periphery (D). Magnifications in the boxed areas with F-actin and vinculin staining are shown on the left. Scale bars represent 10 μm.

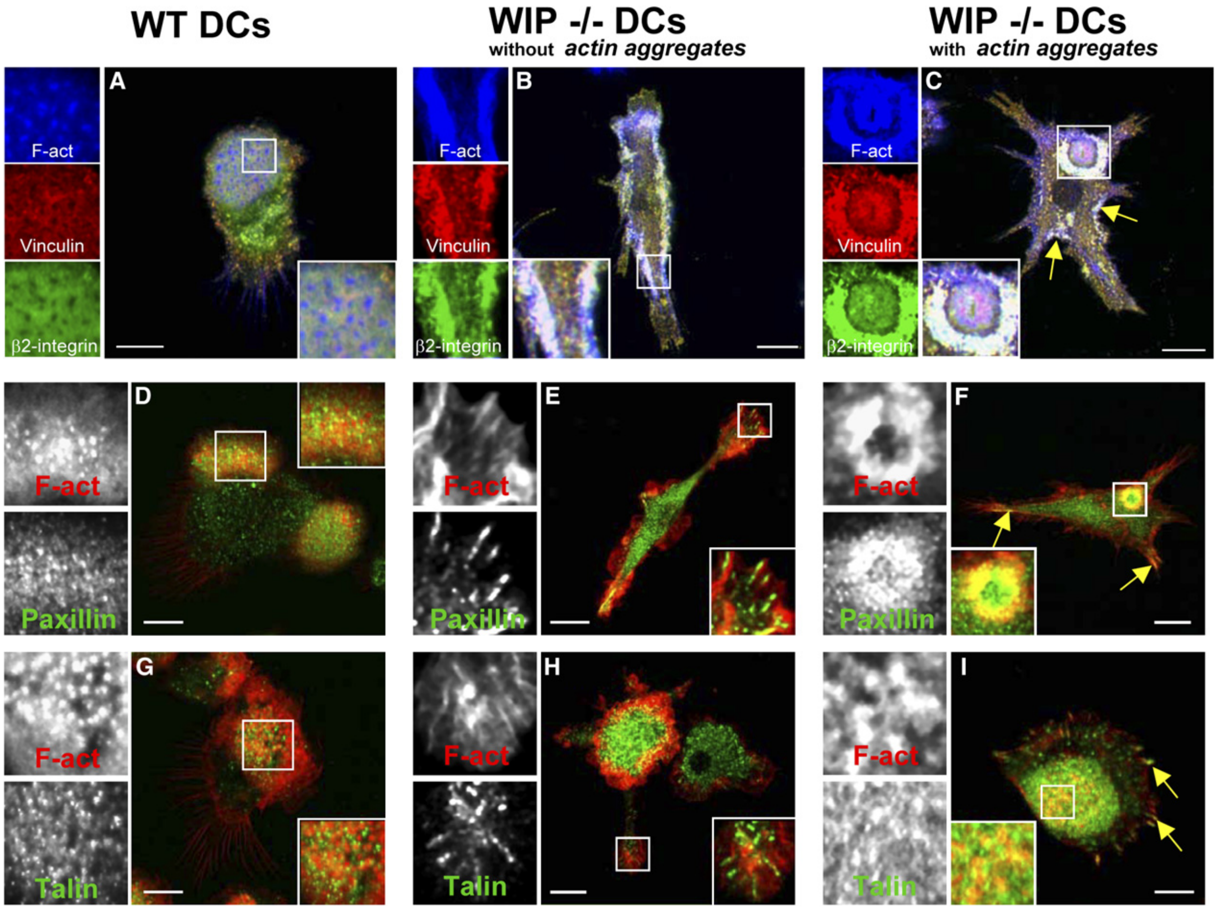

Figure S2. WIP Is Required for the Normal Clustering of β2-Integrins and Integrin-Associated Proteins in DCs

DCs plated on PLL-coated glass coverslips were fixed with 4% paraformaldehyde/3% sucrose, permeabilized with 0.05% Triton X-100, and immunostained to determine the distribution of the corresponding proteins.

(A–C) Distribution of β2-integrins (green) vinculin (red) and F-actin (blue) in wild-type (A) and WIP$^{-/-}$ (B and C) DCs. Lack of WIP resulted in failure to form circular clusters of β2-integrins in DCs. Arrows point at the location of small focal contacts. Magnifications of the boxed areas with F-actin, vinculin, and β2-integrin staining are shown on the left.

(D–I) Distribution of paxillin (green) and F-actin (red) (D–F) and distribution of talin (green) and F-actin (red) (G–I) in WT (D, G) and WIP$^{-/-}$ (E, F, H, I) DCs. Magnifications of the boxed areas with F-actin and paxillin or talin staining are shown on the left. In the absence of WIP, DCs fail to form circular clusters of paxillin or talin, and these integrin-associated proteins form large focal contacts instead. Images are representative of at least three independent experiments. Scale bars represent 10 μm.

anti-CrkL 1:1000 (Santa Cruz Biotechnology, Inc), anti-Nck 1:1000 (BD Biosciences, UK), HRP-conjugated anti-goat IgG 1:1000 (DAKO, UK); HPR-conjugated anti-mouse IgG 1:1000 (DAKO); HRP-conjugated anti-rabbit IgG 1:1000 (DAKO). Calpain inhibition studies were performed with calpain inhibitor I (ALLN), calpain inhibitor II (ALLM), and the cell-permeable calpastatin peptide, all purchased from Calbiochem, UK. As negative control for the effect of the calpastatin peptide, we used the scrambled version of the peptide from Calbiochem. Inhibition of cathepsins and the proteasome was achieved by treatment of DCs with cathepsin inhibitor 1 and proteasome inhibitor 1 (PSI), both purchased from Calbiochem.

Immunofluorescence Microscopy

Freshly prepared 10 μg/ml poly-L-lysine (PLL, Sigma), 10 μg/ml human fibronectin (FN, Sigma) or 10 μg/ml intercellular cell adhesion molecule-1 (ICAM-1, R&D Systems) solutions were incubated over sterile and acid-washed glass coverslips for 1 hr at room temperature before plating cells. 10^5 DCs plated on substratum-coated coverslips were fixed with 4% PFA/3% sucrose for 25 min and permeabilized with 0.05% Triton-X-100/PBS for 10 min. Localization of proteins was achieved by means of an appropriate concentration of antibody diluted in 2.5% BSA/PBS at room temperature for 1 hr. After three PBS washes, samples were incubated with appropriate secondary antibodies diluted in 2.5% BSA/PBS. For localization of filamentous actin, cells were incubated with 0.1 μg/ml Alexa 568-phalloidin (Sigma) for 45 min at 37°C. Coverslips were then washed three times with PBS and twice with distilled H_20 before being mounted in Vectashield mounting medium (Vector Laboratories, UK). Confocal images were obtained with a Zeiss LSM 510 Meta confocal laser scanning head attached to a Zeiss META Axioplan 2 microscope. LSM 510 software was used to collect four sequential images from four separate optical sections in the z axis 0.2 μm apart. The same software was used to obtain merged confocal projections along the z axis via maxima fluorescence values. Images were exported from Database Files.mdb to TIFF files by Zeiss LSM Image Browser and processed with Adobe Photoshop 7.0 software.

Fluorescent Activated Cell Sorting

To prepare samples for FACS analysis, a minimum of 2×10^5 cells per FACS tube were fixed with 4% paraformaldehyde (PFA)/3% sucrose for 20 min. After two washes in PBS by centrifugation (1500 rpm for 5 min), the suspension was incubated in 50 μl of 5% BSA blocking solution to reduce nonspecific binding. The cell samples were centrifuged and resuspended with appropriate amounts of each individual antibody solutions and incubated for 1 hr at room temperature. After three washes with PBS, cells were incubated in a dilution of 1:100 of the corresponding fluorescently tagged secondary antibody followed by three washings in PBS

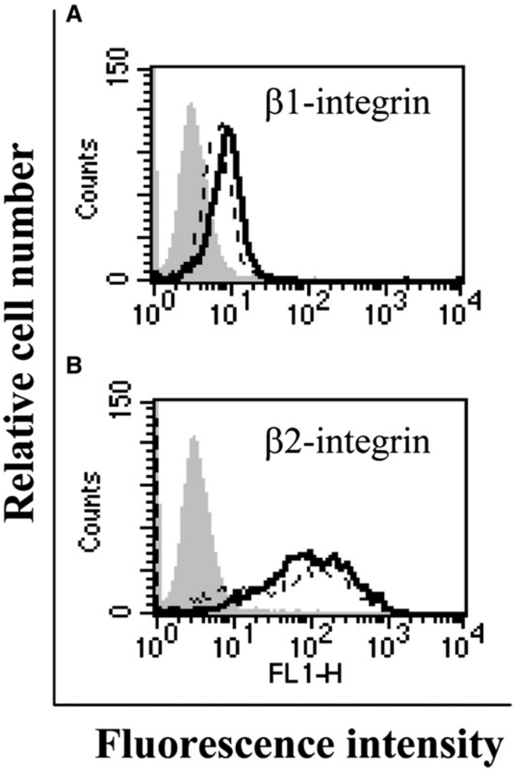

Figure S3. Quantitative Analysis of β1- and β2-Integrin Expression in DC by FACS

2×10^5 DCs were harvested and incubated with β1 or β2 antibodies followed by the appropriate secondary FITC-labeled antibody. Graphs illustrate the distribution of the expression of β1 (A) and β2 (B) integrins in DCs. Shaded histograms represent cells stained with secondary antibody alone, as negative control; full line and dotted line histograms represent the staining of wild-type DCs and $WIP^{-/-}$ DCs, respectively. The histograms show the results of one experiment representative out of three.

before FACS was performed. Controls included cells alone as autofluorescence control, and cells incubated with FITC-conjugated secondary antibodies were used as negative controls for these experiments. The percentage of positive cells was obtained by setting the percentage of negative controls as 2%–5%. In all experiments, 10,000 cells were analyzed. FACS analysis was performed via FACS Calibur with CellQuest software (Becton Dickinson, San Jose, CA). All analyses were carried out a minimum of three independent occasions.

Analysis of Adhesion Turnover

We used interference reflection microscopy (IRM) [S3, S4] to visualize the adhesion-substratum interface of living cells. DCs in complete culture medium were plated on the glass coverslips previously coated with the relevant substratum. Coverslips were mounted and sealed onto viewing chambers in culture medium and held at 37°C. Interference reflection images were collected with a Zeiss Standard 18 microscope with an incident light fluorescence attachment. Exciter and barrier filters were removed from the LP420 reflector and replaced with a narrow band pass filter to isolate the 546 nm line of the mercury arc source. Coverslips with attached cells were observed with a Zeiss 63× Neofluar Antiflex oil immersion objective, NA 1.25. Images were recorded digitally with in-house software and replayed as a movie. Alternatively, podosome turnover in DCs expressing eGFP constructs (see below) was performed by simultaneously visualizing GFP signal and adhesion-substratum interface with a Zeiss LSM META confocal scanning head as described above, and with the 488 nm line of an Argon laser and a 470–500 nm band pass filter to detect the interference reflection signal and a 505 nm long pass filter to detect the eGFP signal. The resulting multi-image TIFF files were processed with customized Adobe Photoshop version 7.0 to threshold as well as to increase contrast and sharpness of the adhesion points of the cells with the substratum. To analyze the persistence of adhesion points, 10 images taken 30 s apart were overlapped via the difference function in Adobe Photoshop. Each image was thresholded to produce black adhesions on white background and then inverted as white adhesions on black background. Next, the image was divided by 10 to obtain dark gray corresponding to adhesions (i.e., 256/10 on the scale of 1–256). The 10 images were then overlapped and reinverted. Finally, a composite image with 10 relevant gray levels was obtained. The lightest gray level represented pixels that were present in one of the ten images (adhesion points last for 30 s), and the darkest gray level represented pixels that were present in 10 out of 10 images (i.e., adhesion points last for 300 s). Therefore, the areas of light gray color pixels represent dynamic adhesions whereas areas of dark gray and black color pixels represent stable adhesions over the selected interval of time. With the histogram function of Adobe Photoshop, we could quantify the percentage of pixels per image corresponding to each gray level, which allowed us to calculate the turnover index by dividing the percentage of pixels present in 9 and 10 of the gray scale map by the percentage present in 1 and 2 of the gray scale map. Turnover index = (% of pixels present in gray level 9+10)/(% of pixels present in gray level 1+2). Thus, a ratio of unstable adhesion over stable adhesion in each live cell was

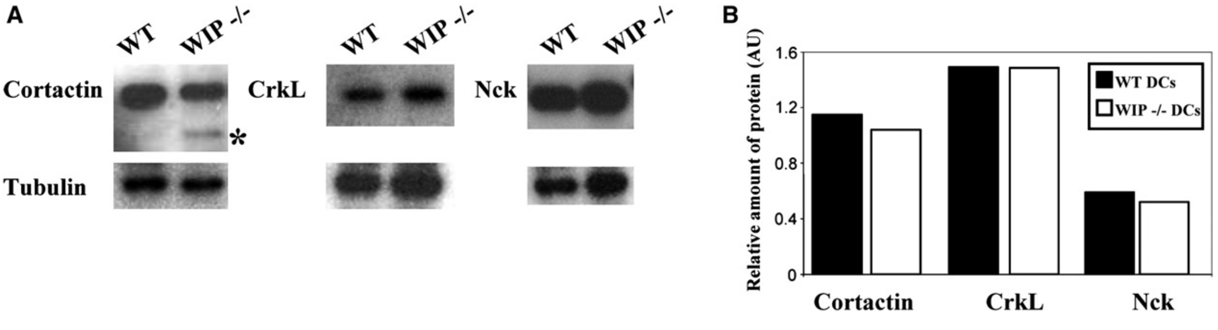

Figure S4. Comparison of Expression of WIP-Binding Partners between Wild-Type and $WIP^{-/-}$ DCs

(A) Total lysates of wild-type (WT) and $WIP^{-/-}$ DCs were resolved by 12% SDS-PAGE, western blotted, and probed with cortactin, Nck, CrkL or tubulin antibodies after stripping according to the experiment. Tubulin showed the loading control in each lane.

(B) Graph shows the quantification of the expression of relative amounts of the proteins detected by immunoblot as shown in (A). The intensity of luminosity (I. L.) and the area of each band in an inverted image was calculated with the histogram function of Photoshop 7.0 and the value of relative amount of protein (AU) was calculated as follows: I. L. × Area (protein to be tested)/I. L. × Area (tubulin). No significant differences were detected in expression of cortactin, Nck, and CrkL between wild-type and $WIP^{-/-}$ DCs. Of note, in cortactin immunoblots we detected an increase in a band of approximately 70 kDa (asterisk) likely to be a calpain-mediated cleavage product previously described [S8].

obtained. The higher value of the turnover index represents the more dynamic of the cell adhesion. Unpaired Student's t test was used to assess the significance of experimental results.

Kymography

IRM image stacks were imported into Mathematica 5.2 where they were processed to normalize image contrast. Two intensity line-scans were then taken for each image in the stack: one was along the largest axis of spread DCs, which display an elongated morphology (in the case of wild-type DCs, this axis corresponds with the direction of cell movement), the other perpendicular to this and centered on a region just behind one end of the axis. Line-scans from each image in the stack were then used to construct kymographs. 1-dimensional line-scans were then converted into a 2-dimensional array for each image stack. The resultant array was then represented as a kymograph with time on the y axis of the image and distance along the intensity profile on the x axis. The first kymograph enabled visualization of protrusive events in the direction of motion, whereas the second kymograph enabled visualization of protrusive events perpendicular to this. Thus, data, such as the rate of protrusion at the leading edge and the number, extent, and frequency of protrusions at the sides of the cell could be determined.

Western Blot

Cell lysates from either untreated or protease inhibitor-treated DCs were obtained by adding Laemmli sample buffer to plated cells. Approximately 20 μg of total cell lysate protein was loaded per lane in a 12% SDS-PAGE gel and subjected to electrophoresis. Proteins were blotted onto PVDF membranes with a Bio-Rad Mini protein II transfer apparatus. Blots were blocked with 5% dried milk/TBS-T for 1 hr at room temperature, incubated with indicated antibodies at 4°C overnight. After three washes with TBS-T, immunoprobed proteins were detected by incubation with horseradish peroxidase-conjugated secondary antibodies at room temperature for 1 hr. After further washes in TBS-T, immunoprobed proteins were visualized by ECL chemiluminescence kit (Amersham, UK), exposed on Hyperfilm ECL (Amersham, UK), and developed with an Imaging Systems Xograph compact X4 developer. Blots were reprobed after treating with stripping buffer at 50°C for 30 min three times.

RT-PCR

Total RNA from 2×10^6 WT DCs and $WIP^{-/-}$ DCs for RT-PCR was prepared with GenElute Mammalian Total RNA kit (Sigma), and Enhanced Avian HS RT-PCR kit (Sigma) was used to subsequently amplify the resulting cDNA. To avoid the possible contamination of DNA, DNase digestion (Amplificationn Grade DNase 1, Sigma) was further performed prior to RT-PCR. The specific oligonucleotide primers designed based on the WASP nucleotide sequence were: forward primer, 5′-GAT GTG AAC AAC CTA GAC CC-3′; reverse primer, 5′-GCA CCC CCC ATA GGT ACA GG-3′. β-actin was used as a control reaction and the primers were: forward primer, 5′-TGC GTG ACA TTA AGG AGA AG-3′; reverse primer, 5′-CTG CAT CCT GTC GGC AAT G-3′. One-Step RT-PCR Reaction was selected as the PCR condition (35 cycles, annealing 55°C) according to manufacturer's instructions. The PCR products were analyzed by running on a 1% agarose gel and subsequent ethidium bromide staining.

Infections of DCs with Lentiviral Vectors

Lentiviral vector stocks were produced in 293T cells by cotransfecting the transfer vector pHR'SINcPPT-SFFV-eGFP-(SEW) or SFFV-eGFP-WASP (SEWW), the envelope plasmid pMD.G, and the packaging plasmid pCMVR8.91, as previously described [S5]. 1.5×10^7 cells were seeded onto 150 cm^2 flasks and transfected with 10 μg DNA envelope, 30 μg DNA packaging, and 40 μg DNA transfer vector by precomplexing with 0.125 μM PEI (22 kDa) for 15 min at room temperature in Optimem. After 4 hr at 37°C, the medium was replaced with fresh DMEM 10% FCS and virus were harvested 48 and 72 hr posttransfection. After filtering through a 0.45 μm-pore-size filter, the virus suspension was concentrated by centrifugation at 50,000 × g for 2 hr at 4°C. The resulting pellet was resuspended in RPMI (Sigma) and stored at −80°C until use. The desired number of DCs were plated in complete culture medium as described above with phenol-free RPMI (Sigma) and lentivirus containing supernatant was added to the cells at an MOI of 100 and incubated for 24 hr. Media was replaced for complete DC culture medium without phenol-free RPMI after 24 hr, and cells were cultured for another 48 hr to allow maximal expression of lentiviral vectors before being used in experiments.

Generation of Mutant WASP Expression Constructs

Human wild-type (WT) WASP was cloned from the FL3 plasmid (gift from J. Derry) into the pEGFP-C1 (Invitrogen) cloning vector, and A134T and R138P point mutations were introduced via the Quikchange protocol (Qiagen). The VCA domain-deleted WASP contruct (δVCA) was generated by PCR with a reverse primer starting at nucleotide 1288 of the WASP gene, and cloned into pEGFP-C1. WT and mutated WASP were then subcloned into the EF-Bos-GST expression vector, containing GST amplified by PCR from the pGEX-2T vector. Mutant and WT WASP in the EF-Bos-GST vector were then sequenced to confirm the presence or absence of point mutations or truncation.

Cell Transfection and Recombinant Protein Production

COS-7 cells (7×10^6) were electroporated with 20 μg of plasmid DNA in 400 μl of special electroporation buffer (120 mM KCl, 10 mM K_2PO_4/KH_2PO_4 [pH 7.6], 2 mM $MgCl_2$, 25 mM HEPES [pH 7.6], 0.5% Ficoll 400) by a Biorad Genepulser II at 250V and 975 μF. After overnight growth, cells were harvested in ice-cold lysis buffer (1% Nonidet P40, 130 mM NaCl, 20 mM Tris-HCl [pH 8.0], 10 mM NaF, 2 mM sodium orthovanadate, 1% aprotinin, 10 μg/ml leupeptin, 1 mM phenylmethylsulfonyl fluoride, 2 mM EDTA, Cytochalasin D 2 μM, Latrunculin B 2 μM), and lysates were clarified by centrifugation at 15,000 × g for 10 min.

Actin Polymerization Assay

The bead-based actin polymerisation assay was modified from an assay previously described [S6, S7]. COS-7 cell lysates (transfected with WT or mutant GST expression vectors or GST alone) were incubated with 75 μL of glutathione sepharose 4B beads (Amersham Biosciences) for 2 hr at 4°C. Beads were then washed with lysis buffer (×3), 0.5 M LiCl, 20 mM Tris (pH 8.0) (×2), 20 mM Tris (pH 8.0) (×1) and stored in 75% glycerol, 10 mM TRIS (pH 8.0), 1 mM EDTA at −20°C. U937 cell lysates were prepared by lysing 1×10^7 cells in 1 ml of actin polymerization buffer (1% Nonidet P40, 130 mM NaCl, 20 mM Tris-HCl [pH 7.4], 1% aprotinin, 10 μg/ml leupeptin, 1 mM phenylmethylsulfonyl fluoride, 1 mM EDTA), and then clarifying as above. This lysate was then supplemented with 5 mM $MgCl_2$, and 1 mM ATP. 250 μl of lysate was then incubated with 8 μl of GST-WASP-coated sepharose beads on a rotor for 1 hr at room temperature. A negative control was performed with 2 μM cytochalasin D added to the lysate to inhibit actin polymerization. The beads were then washed with actin polymerization buffer (×2) prior to pelleting and mixing with NuPAGE LDS Sample Buffer and reducing agent (Invitrogen) and heating to 70°C for 10 min. Samples were resolved by electrophoresis with 4%–12% Nupage Bis-Tris gel and MOPS buffer (Invitrogen). Blots were stained with B-14:sc-138 monoclonal mouse GST mAb (Santa Cruz Biotech) (>65 KDa and <35 KDa) or rabbit anti-actin affinity isolated antibody (Sigma A2066) (35–65 KDa). HRP-conjugated rabbit anti-mouse and goat anti-rabbit antibodies (Dako) were used, respectively, and the ECL Western detection system (Amersham) was used for detection. Images were captured with the Uvichemi super-cooled camera and image documentation system. The assay was repeated four times.

Supplemental References

S1. Anton, I.M., de la Fuente, M.A., Sims, T.N., Freeman, S., Ramesh, N., Hartwig, J.H., Dustin, M.L., and Geha, R.S. (2002). WIP deficiency reveals a differential role for WIP and the actin cytoskeleton in T and B cell activation. Immunity *16*, 193–204.

S2. Snapper, S.B., Rosen, F.S., Mizoguchi, E., Cohen, P., Khan, W., Liu, C.H., Hagemann, T.L., Kwan, S.P., Ferrini, R., Davidson, L., et al. (1998). Wiskott-Aldrich syndrome protein-deficient mice reveal a role for WASP in T but not B cell activation. Immunity *9*, 81–91.

S3. Curtis, A.S.G. (1964). The mechanism of adhesions of cells to glass: a study by interference reflection microscopy. J. Cell Biol. *20*, 199–215.

S4. Dunn, G.A., and Jones, G.E. (2004). Cell motility under the microscope: Vorsprung durch Technik. Nat. Rev. Mol. Cell Biol. *5*, 667–672.

S5. Zufferey, R., Nagy, D., Mandel, R.J., Naldini, L., and Trono, D. (1997). Multiply attenuated lentiviral vector achieves efficient gene delivery in vivo. Nat. Biotechnol. *15*, 871–875.

S6. Cory, G.O., Cramer, R., Blanchoin, L., and Ridley, A.J. (2003). Phosphorylation of the WASP-VCA domain increases its affinity for the Arp2/3 complex and enhances actin polymerization by WASP. Mol. Cell *11*, 1229–1239.

S7. Fradelizi, J., Noireaux, V., Plastino, J., Menichi, B., Louvard, D., Sykes, C., Golsteyn, R.M., and Friederich, E. (2001). ActA and human zyxin harbour Arp2/3-independent actin-polymerization activity. Nat. Cell Biol. *3*, 699–707.

S8. Perrin, B.J., Amann, K.J., and Huttenlocher, A. (2006). Proteolysis of cortactin by calpain regulates membrane protrusion during cell migration. Mol. Biol. Cell *17*, 239–250.
